# Auto-assessment of assessment: A human-in-the-loop AI framework addressing policy gaps in academic assessment

**DOI:** 10.1371/journal.pone.0346815

**Published:** 2026-04-15

**Authors:** Wasiq Khan, Luke Topham, Nathan Jones, Peter Atherton, Raghad Al-Shabandar, Hoshang Kolivand, Iftikhar Khan, Abbas Alatrany, Abir Hussain

**Affiliations:** 1 Liverpool John Moores University, School of Computer Science and Mathematics, Liverpool, United Kingdom; 2 Advisory Office, Office of the Prime Minister, Baghdad, Iraq; 3 University of Leicester, School of Computer Science, Leicester, United Kingdom; 4 Universityof Sharjah, College of Engineering, Sharjah, United Arab Emirates; Federal University of Paraiba, BRAZIL

## Abstract

Education is being transformed by rapid advances in Artificial Intelligence (AI), including emerging Generative Artificial Intelligence (GAI). Such technology can significantly support academics and students by automating monotonous tasks and making personalised suggestions. However, despite the potential of the technology, there are significant concerns regarding AI misuse, particularly by students in assessments. There are two schools of thought: one advocates for a complete ban on it, while the other views it as a valuable educational tool, provided it is governed by a robust usage policy. This contradiction clearly indicates a major policy gap in academic practices, and new policies are required to uphold academic standards while enabling staff and students to benefit from technological advancements. We surveyed 117 academics from three countries (the UK, the UAE, and Iraq) and identified that most academics retain positive opinions regarding AI in education. For example, the majority of experienced academics do not favour complete bans, and they see potential benefits of AI for students, teaching staff, and academic institutions. Importantly, academics specifically identified the particular benefits of AI for autonomous assessment (71.79% of respondents agreed). Therefore, for the first time, we introduce a novel AI framework for evaluating students’ work (e.g., reports, coursework, etc.) within a human-in-the-loop assessment process, in which automated grade suggestions are generated and subsequently reviewed by qualified instructors based on students’ knowledge and in-depth understanding of the submitted content. The survey results and evaluation outcomes further highlight a significant lack of awareness of modern AI-based tools among experienced academics, a gap that must be addressed to uphold educational standards.

## 1. Introduction

AI has tremendously transformed the educational field. Integrating AI technology in the education sector has shaped and fostered the traditional learning method to be more interactive and effective [1]. This intersection of Artificial Intelligence and education, commonly referred to as Artificial Intelligence in Education (AIEd), involves the design, application, and study of AI techniques to enhance teaching, learning, and educational management. AIEd encompasses systems that adapt to learners’ needs, automate assessment processes, provide intelligent tutoring, and support data-driven educational decisions. AI plays a crucial role in supporting higher education by facilitating the learning process for students through providing a personalised learning environment and promoting student engagement [2].

Large Language Models (LLMs), such as ChatGPT, have recently demonstrated significant advances in human-like text generation. Their versatility and diverse applications, including question and answering, text classification, text generation, inference, and more [3], have made a significant impact in domains such as public health [4], customer relations [5], finance [6], and education [7]. Despite the promise of the technology, there remains significant controversy regarding its use in academia, with many suggesting it should be banned [8].

Furthermore, the lack of clear policies, guidelines, and frameworks prevents LLMs from being harnessed in academia [9]. Policymakers are struggling to keep up with the rapid development of LLMs and other Artificial Intelligence technologies. This is exacerbated by a lack of understanding of academics’ experience and perceptions of LLMs due to insufficient relevant empirical studies [9]. This gap must be bridged while ensuring that academics’ voices are heard in the policy development process to help shape the future of LLM use in academia.

Similarly, most institutions are developing guidelines without collaborating with other institutions. Therefore, there is a lack of consistency in existing policies between institutions [10]. Moreover, a lack of consistency exists between departments, faculties, courses, and even modules within institutions [10]. For example, some institutions encourage module leaders to set the rules and guidelines for LLM use for each assignment [10]. This results in students being allowed to use LLMs in specific assessments, facing restrictions in some assessments, and being subject to a total ban in others. A lack of consistency results in confusion for academics and students and makes it difficult to compare work across modules, courses, and institutions. Despite LLMs’ potential benefits, many academics are concerned with their misuse, such as students submitting generated text as their own during assignments. Such concerns are exacerbated by difficulties in differentiating human-written text from LLM-generated text [11]. Such challenges and concerns have led many institutions to ban ChatGPT and other tools [12]. However, such bans are difficult to enforce and result in some students gaining an unfair advantage [12]. Similarly, complete bans prevent students from benefiting from the valuable tools available and pose a barrier to their learning skills, which will benefit them in the workplace [13]. The aforementioned contradictory opinions clearly indicate major policy gaps in academic institutions that need to be addressed to maintain educational standards across the globe. In this regard, we survey experienced academics to investigate multiple perspectives with the following Research Questions (RQs):

*RQ1: What are the existing policy gaps in the assessment of student work within academia regarding the emergence of generative AI technologies?*

*RQ2: Would AI be helpful in automatically ranking students’ work submissions based on their understanding/knowledge of the subject matter, rather than solely for detecting ChatGPT (GAI) usage?*

The remaining manuscript is organised as follows: the relevant literature is reviewed in Section 2; our methodology, including the data collection method and proposed framework, is described in Section 3; detailed results, framework evaluation, and discussion are provided in Section 4, and the conclusion and suggestions for future work are presented in Section 5.

## 2. Related works

The rapid integration of GAI into educational contexts challenges existing paradigms of assessment, academic integrity, and student learning development [1]. While much of the existing literature focuses on identifying and mitigating instances of AI misuse, emerging perspectives advocate for a shift towards conceptual frameworks that integrate AI as an educational partner rather than a threat [9]. Recent literature highlights a tension between efforts to detect and prevent AI-generated student work and emerging perspectives that advocate for integrating AI tools into authentic learning and assessment practices [9]. Debates surrounding assessment design, academic integrity, and the ethical use of AI technologies increasingly point to the need for more proactive and sustainable approaches in higher education contexts. Our study builds on this body of work to propose an alternative framework for AI-driven autonomous assessment, informed by empirical insights from academic practitioners.

Several frameworks and approaches have been proposed to assist in identifying cases in which students submit LLM-generated work as their own. For instance, [14] recommend examining linguistic patterns and irregularities, such as repetitive phrasing, grammatical inconsistencies, or reduced language quality that may arise from limitations in LLMs. The study also highlights the importance of verifying the accuracy of references and citations, because LLMs frequently generate non-existent or incorrect sources. In addition, assessing the originality of submissions is suggested, given that LLM-generated content typically lacks human-level creativity. Factual inaccuracies or inappropriate content may further indicate the use of LLMs, and a range of automated detection tools has been developed to support educators by identifying textual patterns and anomalies.

Although the suggested approaches have shown some success, several limitations affect their practicality. For example, such approaches are time-consuming for already stretched academic staff. Moreover, the suggested approaches are designed to identify LLMs in their current or previous forms. At the current rate of progress, future LLMs will likely render these approaches useless. Even when academics follow such approaches and identify suspected cases of academic misconduct, establishing conclusive proof and enforcing appropriate sanctions remain challenging [15].

Alternatively, many institutions suggest altering the style of assessments to make them less susceptible to the misuse of LLMs. For example, [16] suggests interactive assessment activities such as presentations or group discussions to prevent or minimise the potential use of LLMs. Some suggest that such assessment methods may promote independent learning and critical thinking, particularly as there is an opportunity to encourage students to elaborate on or defend specific points [17]. Similarly, “authentic assessments” are promoted, where students are assessed on real-world tasks [17]. Despite the promise of open-ended and authentic assessments, such approaches require significant effort and academic supervision and are not appropriate for all assessments [18].

These varied approaches reflect underlying tensions in assessment theory between traditional summative evaluation and more authentic, formative practices that emphasise critical thinking and real-world relevance. This tension also highlights the challenge of balancing academic integrity with fostering autonomous, technology-enhanced learning.

Thus far, most proposed frameworks focus on limiting opportunities for LLM use to minimise the risk of misuse. However, the tools are publicly available and provide many potential benefits, such as improved working efficiency. Therefore, it is logical to assume that workplaces will seek to adopt such tools. Consequently, by preventing students from learning to use such tools effectively, such frameworks may impede their learning and improve their employability.

in addition to the frameworks discussed in the literature, it is important to consider the students’ experiences and perceptions. According to [19], students are generally attracted to AI tools such as ChatGPT and show improved engagement and motivation. However, they suggest that students must be trained to enhance their prompt engineering skills to achieve better results from the tools [19]. Moreover, the need for training is also highlighted [20], who suggested that students are more likely to trust AI tool results unquestioningly. Therefore, they suggest improving students’ critical thinking skills and encouraging them to analyse the results’ reliability and verify their correctness [20]. Furthermore, in cases where students have been trained to use AI tools appropriately and within the rules of assessment, students have demonstrated a recognition of the value of the ethical use of such tools [21]. However, many students are reluctant to use AI tools as they perceive them as cheating [21].

In addition to the students’ experiences and perceptions, it is also essential to consider those of academics and teachers. Like the students’ positive perceptions, teachers also view AI tools as beneficial to their teaching [7], particularly in lesson planning, assessment, and the preparation of learning materials. However, they report concerns regarding the accuracy of information provided, bias, and a lack of human interaction [22]. Similarly, some teachers also highlight the need for students to learn to ethically use AI tools to aid their learning and to prepare them for the future, as they recognise that AI tools are not likely to disappear [23]. Despite many teachers perceiving the ethical use of AI tools as beneficial, many teachers still perceive them as a threat. In addition to the aforementioned academic misconduct concerns, some academics are concerned that using AI tools may decrease students’ skills, for example, in analytical writing [24].

Consequently, an essential consideration is the ethical use of AIEd, as it raises important questions about fairness, transparency, and the impact on academic integrity. Ethical frameworks in AIEd tend to be context-dependent and can vary significantly across countries, regions, and domains [25]. That said, there are AI policies like UNESCO’s, whose global perspective aims to establish adherence to agreed ethical norms [26]. Studies are drawing on growing evidence that LLM can generate high-quality writing for many purposes and in a way that can be passed on to humans. AI writing is hard to detect via anti-plagiarism software and is often of superior quality to students’ own output, including reflective writing [9]. Crompton and Burke’s meta-systematic review [27] concluded that higher education uses AI widely for student assessment and evaluation but needs to be managed more ethically, collaboratively, and rigorously [27]. Universities are publishing assessment policies in light of GAI, but education is only beginning to allow their policies to be informed by viable conceptual frameworks [28]. While institutions themselves have responsibilities regarding AI and ethics, the prevalence of LLMs in students’ work also raises questions about students’ need to develop a balanced and critical approach to how they use AI [29].

There are ethical concerns in the literature regarding predictive AI and GAI. Predictions made by AI can be the product of models that have been trained on biased data [30]. In terms of GAI, while LLMs, like ChatGPT, are benefiting students and teachers, there are concerns over bias in both the output of ChatGPT and the broader society. Furthermore, the functionality of some AI has been criticised, such as the risk of hallucinations caused by semantic limitations or biased programming [31]. The ethical dimensions of AIEd require ongoing refinement and augmentation to ensure student and institutional data privacy and minimise bias [32]. Similarly, studies have shown a need for re-skilling educators and addressing the risk of deskilling students [13].

The notion of authenticity is being problematised. Previous studies have perhaps viewed AI as an external assistant to a passive receiver, failing to acknowledge the hybrid, collaborative nature of knowledge production [28]. AI content, then, has a ‘social life’ in terms of its conception, production, dissemination, context, and multiple uses [33]. Indeed, more recent studies have recommended more human-centred approaches to developing LLMs. Such approaches could acknowledge the synergies between human capabilities and data [34]. In addition, a focus on the complementary attributes of humans and AI could be a catalyst for innovation [35]. Moreover, some studies have concluded that AI can make education more human, not less, and can also promote well-being if used alongside positive psychology [36]. In contrast, the literature reports evidence of that human intervention has a damaging effect on machine learning [34].

AI policy should focus on ethics, prioritising the development of AI solutions that respect human rights and safety. The development of AI technologies must be governed by a legal and regulatory framework. This framework aims to ensure the transparency and accountability of the AI system [37]. For instance, the governance ethical framework can contain laws and regulations, rules, and ethical committees that focus on ethics, prioritising the development of AI solutions that respect human rights and safety [38].

Another central consideration is how learning will be evaluated in classrooms that use AIEd. The assessment of student learning is being augmented by improved AI algorithms, for example, in the domains of predictive learning analytics [7]. AI can aid autonomous learning via students’ own questions [39], and assist with answers to open-ended questions [40]. The assessment of students’ work can generate real-time data, which AI can evaluate, to help with programme design [41].

Earlier systematic reviews on AI and assessment recognised an absence of discussion of underlying pedagogies that may lead to automated assessment and recommended further research and teacher training [42]. Indeed, ongoing teacher training is recognised as necessary to enable educators to harness new technologies to enhance learning outcomes [43].

Some recent literature has examined the architecture of AIEd on a more granular level, for example, the inaccuracies of static modelling versus the responsiveness and agility of dynamic modelling [7]. Another benefit of dynamic modelling is the accuracy with which systems can alert educators to students who may be at risk of underachieving and, therefore, facilitate timely interventions [7]. More recent literature on LLM builds on studies into predictive modelling techniques from the mid-2010s [44].

This dynamism has informed more recent studies into using LLM to answer open-ended questions, though the range of studies is emerging, and the datasets can be sparse [45]. The limited nature of datasets in AI-related studies is a prominent theme in the literature [46]. While some authors may recommend “massive volumes of standardised datasets” [46], others are more circumspect about the preponderance of big data [43]. Predictive AI can misrepresent students’ future behaviours, and the data can be mined as part of a ‘digital capitalism’ characterised by economic, political, and cultural accumulation and exploitation of data as capital [33].

Despite increasing recognition of GAI’s pervasive role in education, much of the existing scholarship remains fragmented, addressing detection strategies, ethical concerns, or pedagogical adaptations in isolation. There is a need for integrated conceptual frameworks that account for the dynamic interplay between AI capabilities, assessment practices, and ethical considerations. Our study addresses this gap by empirically exploring institutional perspectives across various contexts and proposing a conceptual model for AI-driven autonomous assessment that reconciles technological advantages with educational integrity.

## 3. Methodology

**Fig 1:**
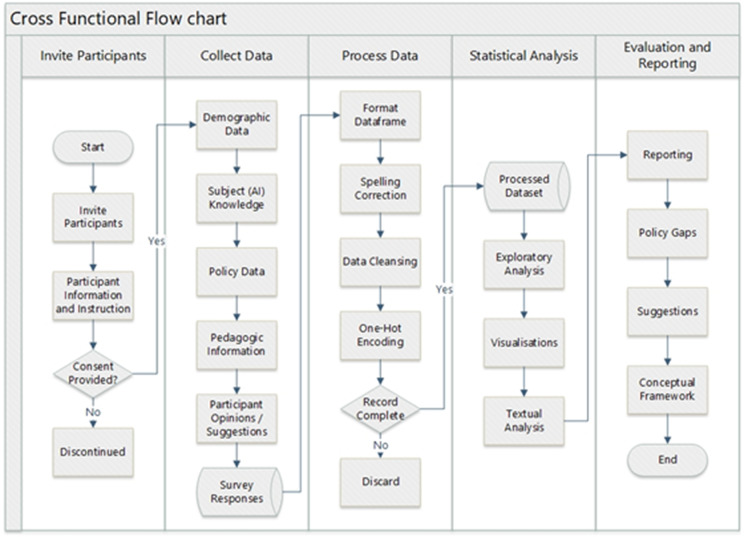
A workflow diagram provides an overview of the project, including participant invitation, data collection, data processing, statistical analysis, evaluation, and reporting.

Given the aforementioned RQs, the proposed methodology is shown in Fig 1. It comprises two major components: i) surveying the expert academics and analysing the outcomes to investigate the RQs, and ii) implementation of a new framework for the autonomous grading of students’ works along-with quality assurance and human oversight. Feedback from experts is collected to identify common themes, patterns, and areas of consensus or disagreement, particularly to investigate the proposed RQs. Furthermore, we synthesise experts’ opinions to identify key strengths and weaknesses of the proposed conceptual framework in terms of its acceptability and validation for the student assessments. We surveyed 117 academic experts (between 17/04/2024 and 16/05/2024) associated with teaching and learning (T&L) and related administrative roles (e.g., programme leaders, T&L policy managers) to gather information concerning policy matters and investigate the proposed RQs. Survey questions include awareness, policy on GAI, suggestions, acceptance of the proposed AI-based auto-assessment, and other challenges due to the emergence of GAI tools. A detailed list of questions and responses is available in the supplementary material (survey dataset).

Fig 1 provides an overview of the project’s data collection, processing, analysis of responses, and reporting. Potential participants who accept the invitations to contribute to the questionnaire are provided with the necessary information to provide informed consent. Only participants who provided informed consent were allowed to complete the questionnaire. The data collection for the questionnaires sought to gather participant’s knowledge, policy data, pedagogical information, opinions, and suggestions regarding their experience with generative AI in assessments. The collected data was stored and processed in preparation for statistical analysis, including exploratory analysis, visualisation, and textual analysis. The project concludes with the development of a conceptual framework aimed to address the policy gaps and suggestions identified during the analysis.

### 3.1. Questionnaire

The University Research Ethics Committee (UREC) at Liverpool John Moores University (LJMU) provides ethical approval for the collection and processing of the questionnaire data (Reference: 24/cmp/001). Educational staff, including lecturers, management, and administration staff, were recruited internationally to complete a questionnaire.

The data was collected using Jisc Online Surveys, a platform widely adopted in UK academia for research data collection. Participants were recruited through professional academic networks, institutional mailing lists, and targeted outreach, ensuring a diverse representation of roles and perspectives across the UK, the UAE, and Iraq. The sample includes academics involved in teaching and assessment, management and policy, admin, and more. While the sample size (n = 117) reflects the responses obtained within the practical constraints of the study timeline, it is comparable to similar studies in this field [47]. The questionnaire contained the following questions:

- How familiar are you with ChatGPT, an AI-powered language model used for generating text? (Very familiar/ Somewhat familiar/ Not familiar)
- Have you encountered instances where students have used ChatGPT or similar AI tools to assist with their coursework or assignments? (Yes/ No)
- In your opinion, how has the availability of ChatGPT affected the originality of students’ work? (Increased originality/ Decreased originality/ No noticeable impact)
- How would you rate the overall quality of students’ work when ChatGPT is used as a tool? (Improved/ Declined/ No change)
- How effective is your institution at detecting AI-generated content in students’ work? (Very effective/ Somewhat effective/ Not effective)
- In your opinion, should students be allowed to use AI-generated content in their work/assessments? (Yes/ No)
- Does your institution have specific policies or guidelines addressing the use of AI tools like ChatGPT in student work? (Yes/ No)
- What strategies, if any, does your institution employ to mitigate the impact of AI tools on assessment integrity? (Check all that apply) (Education on academic integrity/ Plagiarism detection software/ Manual review of suspicious submissions/ Specific guidelines on citation and referencing/ Other (please specify))
- To what extent are students aware of the implications of using AI tools like ChatGPT in their coursework? (Highly aware/ Somewhat aware/Not aware)
- What challenges, if any, have you encountered in assessing students’ work in the presence of AI tools like ChatGPT? (Open text field)
- Do you have any suggestions for improving the assessment process in light of the prevalence of AI tools like ChatGPT? (Open text field)
- Could AI be used to automatically rank students’ work submissions based on their understanding/knowledge of the subject matter, rather than solely for detecting ChatGPT usage? (Somewhat aware/ Not aware)
- What strategies, if any, does your institution employ to mitigate the impact of AI tools on assessment integrity? (Select all that apply) (Education on academic integrity/ Plagiarism detection software/ Manual review of suspicious submissions/ Specific guidelines on citation and referencing/ Other (please specify)
- Demographic Information: (Role in academia/ Country of work/ Institution type (University, college, school))

### 3.2. Dataset

A primary dataset is collected following the questionnaire presented in Section 3.1. A total of 117 responses to the questionnaire were collected (from 17/04/2024–16/05/2024). Table 1 shows the distribution of responses by country: 43.36% from Iraq, 34.19% from the UK, 16.24% from the UAE, and 4.42% from other countries not listed. Similarly, Table 2 presents the number and proportions of responses for each academic role. The vast majority are involved in teaching & assessment (89.74%), with the remaining involved in management & policy matters (5.13%), admin (1.71%), or other roles (3.42%). Detailed responses from the participants are available in the S1 Dataset.

**Table 1:** The number and proportions of questionnaire responses for each country.

Country	Number	Proportion (%)
Iraq	49	43.36
UK	40	34.19
UAE	19	16.24
Other	5	4.42

**Table 2:** The number and proportions of questionnaire responses for each academic job role.

Role	Number	Proportion
Teaching & Assessment	105	89.74
Management & Policy Matters	6	5.13
Admin	2	1.71
Other	4	3.42

### 3.3. Data processing

The survey outcomes are stored in a secure repository at LJMU. Questions 1–9 are multiple choice and, therefore accept only valid responses via a Microsoft Forms interface, which enforces the relevant rules. Thus, quality assurance (QA) is automated and preventative. Moreover, the multiple-choice responses are quantitatively encoded for ease of processing (e.g., on a scale of 1–5). The coded responses are then tabulated for analysis and visualisation. For example, pie charts and bar charts are used to show the proportions of each response and provide a relative comparison among responses.

However, the remaining questions contain open fields that require retrospective QA measures. Therefore, open fields were corrected for spelling using spellcheck software. Correcting spelling is necessary for automated analysis, for example, when calculating the number of responses for each country. Additionally, relevant distribution visualisations, such as pie charts, were created to display the numerical proportions of each response to the questions.

For the qualitative analysis of these open-ended questions, thematic analysis was conducted using Latent Dirichlet Allocation (LDA). LDA is a topic modelling technique that identifies clusters of words that frequently co-occur, allowing us to identify common themes and concerns across responses. The probabilistic model underlying LDA is outlined in Equation 1, where α and β are corpus-level parameters sampled when processing the open response corpus, $\theta_{d}$ represents document-level topic distributions, and $z_{dn}$ and $w_{dn}$ are word-level variables sampled for each word in the response [47]. For a detailed mathematical explanation of LDA, refer to [47].


$$p\left(D|\alpha ,\;\beta \right)=\;\prod_{d=\text{1}}^{M}\int p\left(\theta_{d}|\alpha \right)\;\left(\prod_{n=\text{1}\;}^{N_{d}}\sum_{z_{dn}}p(z_{dn}|\theta_{d})p(w_{dn}|z_{dn},\;\beta )\right)d\theta_{d}$$ (1)

### 3.4. Statistical analysis

Chi-square tests are used to assess the significance of dependence among various categorical variables collected. The Chi-square test is an established statistical method to determine the significance of dependence between two categorical variables. In this study, we utilise the Chi-square test to statistically assess whether the acceptability of AI-based tools for the auto-assessment, as well as the use of AI-based tools by the students, are associated with other factors (e.g., familiarity with AI, institutional policy, etc.). The formula for the Chi-square test is presented in Equation 2 [48], where $x^{\text{2}},\;O^{i},$ and $E^{i}$ are the Chi-square statistic, observed value, and expected value, respectively. The outcomes from a Chi-square test primarily include:

**Chi-square statistic (χ2):** This test statistic is calculated based on the differences between observed and expected frequencies. A higher value indicates a more significant divergence between the observed and expected data.

**p-value:** The probability that the observed differences occurred by chance. If the p-value is less than a chosen significance level (we used the standard one: 0.05), it suggests that the difference between observed and expected values is statistically significant.

**Degrees of Freedom (df):** This is based on the number of categories in the variables.


$$x^{\text{2}}=\;\sum \frac{{\left(O_{i}-E_{i}\right)}^{\text{2}}}{E_{i}}$$ (2)

Another important metric is prevalence, as defined in Equation 3. It refers to the proportion of respondents within the entire group of respondents who hold a particular belief (i.e., have chosen a particular response to a Multiple-Choice Question (MCQ)). It is particularly useful for understanding opinions about AIEd and their prevalence in the academic community.


$$Prevalence=\frac{\#\;of\;respondents\;selecting\;choice}{\#\;of\;total\;respondents}$$ (3)

Detailed outcomes from above statistical methods are presented in the results (Section 4).

### 3.5. Proposed framework

**Fig 2:**
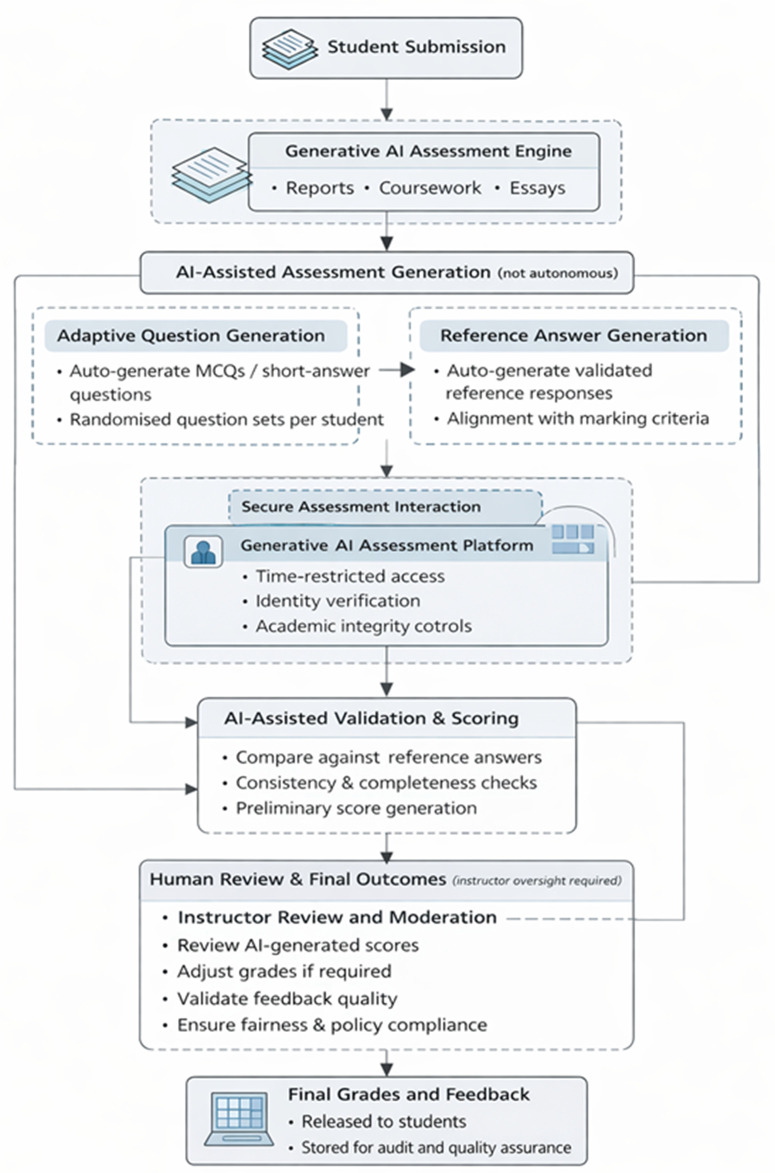
The proposed framework for auto-assessing students’ work submissions comprises GAI, a rule-based algorithm, and human intervention.

We introduce a new framework in Fig 2, for automatically assessing students’ work along-with human oversight for the academic quality and integrity assurance of the process. Students’ original work submissions (e.g., code, reports, etc.) are forwarded to the GAI model (e.g., Ollama in this work). The model randomly generates ‘*N’* number of MCQs from the submitted work, along with the associated correct answers. The auto-generated MCQs can be then forwarded to institutional repositories or Virtual Learning Environments (e.g., Canvas, Blackboard, etc.), where students will be given a preset timeline to respond to the MCQs. These responses are automatically compared with the auto-generated correct answers to produce a grade for the corresponding work. Descriptive feedback is generated based on the student’s responses and their scores. Then, the usual administrative and quality control processes can be followed, such as assessment moderation and the delivery of grades and feedback to students. Appendix 1 (Figures A1-A3 in S1 Walkthrough) demonstrates a prototype workflow and key operational visualisations of the proposed framework.

Similarly, Algorithm 1 describes the logic relating to the proposed automated assessment of assessments (i.e., students’ work submissions). The process begins when students submit work via existing methods, such as a VLE. The submission is then forwarded to a GAI model (Ollama). The GAI model then generates MCQs from the submitted work by extracting key concepts from the submission.

To ensure the quality and fairness of AI-generated MCQs, several safeguards are incorporated within the framework. Firstly, the MCQs undergo validation by a human expert (e.g., an academic or teacher) to detect and correct any factual inaccuracies or biased content that the GAI model may introduce. This human-in-the-loop step mitigates risks associated with hallucinations or biased training data. Secondly, the random generation of multiple MCQs from each submission helps diversify question coverage, reducing the impact of any single biased or erroneous question. Additionally, ongoing monitoring and periodic audits of the question banks will be conducted to identify and address further systemic biases or recurring errors. These measures aim to uphold assessment integrity and fairness while leveraging the benefits of AI-driven question generation. Likewise, correct answers are generated for the MCQs. The MCQs can then be sent to the students via an institutional repository or VLE. Students may then submit their answers, which are compared to the correct answers for grading. Lastly, the GAI model, along with a human expert, generates feedback based on the returned answers and their correctness.

**Algorithm 1** **Implementation steps of the proposed automated assessment framework**

**A.Receive Student Submission**

**Input**: Original student work (code, report, etc.)

**Action**: Student submits work through a designated portal or VLE.

**Output**: Submission is forwarded to the GAI model.

**B.Generate MCQs from Student Submission**

**Input**: Student’s submitted work.

**Action**: The GAI model (e.g., ChatGPT) analyses the submission. **Process**:

•Extract key concepts from the submission.

•Randomly generate N number of Multiple-Choice Questions (MCQs) based on these concepts.

•Generate correct answers for the MCQs.

**Output**: A set of N MCQs and their correct answers.

**C.Forward MCQs to the Institutional Repository/VLE**

**Input**: Generated MCQs and answers.

**Action**: The MCQs are uploaded to the VLE (Canvas, Blackboard, etc.).

**Output**: Students access the MCQs within a preset timeline.

**D.Collect Student Responses**

**Input**: Student responses to the MCQs within the VLE.

**Action**: Students submit their answers.

**Output**: Responses are stored for comparison.

**E.Compare Responses with Correct Answers**

**Input**: Student responses and auto-generated correct answers.

**Action**: Responses are automatically compared with the correct answers using predefined grading algorithm.

**Output**: Raw grade based on correct/incorrect answers.

**F.Generate Descriptive Feedback**

**Input**: Student responses and achieved scores.

**Action**: The AI model generates feedback based on the correctness of answers, while the Human

**G.**Expert performs the overall validation.

## 4. Results and discussion

This section presents and discusses two complementary sets of results that together evaluate the feasibility, acceptance, and practical implications of AI-supported assessment in higher education. First, we report the findings of a cross-national survey of academics conducted prior to the development of the proposed framework, which captures educators’ perceptions, concerns, and expectations regarding the use of AI in assessment and teaching practices. These results provide insight into prevailing attitudes toward AI adoption, including levels of acceptance, perceived benefits, and areas of resistance.

Second, we present the outcomes of an empirical evaluation conducted after the implementation of the proposed AI framework, in which the system was tested with a cohort of 20 students and instructors. This phase focuses on the practical performance of the framework, its usability, and its perceived effectiveness within a human-in-the-loop assessment process. Together, these two sets of results enable a comprehensive discussion that links academic perceptions with real-world deployment, allowing for a critical examination of both anticipated and observed impacts of AI-assisted assessment.

### 4.1. Survey results: Academic views on AI in education

**Fig 3:**
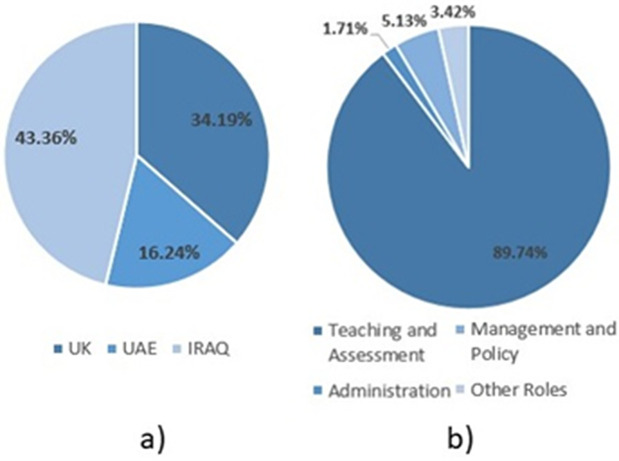
Pie Charts Showing the Diversity of Participants, Includinga) Country of Work and b) Job Role Type.

Table 3 presents the prevalence of each of the possible responses from the MCQs from the survey described in Section 3.1. A total of 117 responses were collected from educational staff in the UK (34.19%), UAE (16.24%), Iraq (43.36%), and others (4.42%), as shown in Fig 3. Most responders were primarily involved in teaching and assessment (89.74%), with the remaining responsible for administration (1.71%), management and policy matters (5.13%), and other roles (3.42%), as shown in Fig 3.

**Table 3:** The prevalence of the multiple-choice question responses from the collected survey, containing 117 participants.

Question	Response	Prevalence (%)
Country of work	Iraq	43.36
	UAE	16.24
	UK	34.19
	Other	4.42
Role in Academia	Teaching and assessment	89.74
	Admin	1.71
	Management and policy matters	5.13
	Other	3.42
LLM Familiarity	Very familiar	43.59
	Somewhat familiar	46.15
	Not Familiar	10.26
ChatGPT Noticed	Yes	81.20
	No	18.80
Originality Affected	Increased	18.80
	Decreased	64.96
	No noticeable impact	16.24
Quality Affected	Improved	37.61
	Declined	47.86
	No change	14.53
Detecting AI-Generated Content	Very effective	11.97
	Somewhat effective	42.74
	Not effective	45.30
ShouldAI-generated content be allowed	Yes	54.70
	No	45.30
Does your institution have policies	Yes	47.86
	No	52.14
Student awareness	Highly aware	22.22
	Somewhat aware	65.81
	Not aware	11.97
Institutional strategies	Education on academic integrity	53.85
	Plagiarism detection software	60.68
	Manual review of suspicious submissions	47.86
	Specific guidelines on citation and referencing	39.32
	None	8.55
	Other	1.71
Use of AI for auto-assessment	Yes	71.79
	No	28.21

**Fig 4:**
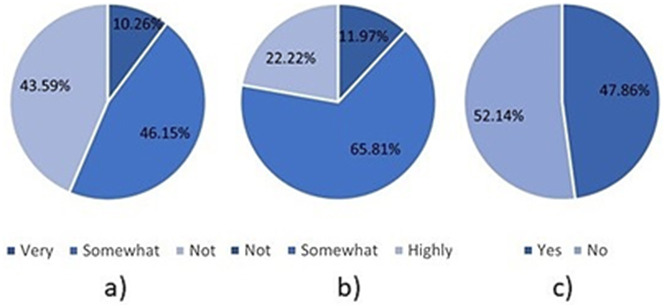
Limitations identified in terms of the awareness of AI in education. **a)** LLM familiarity, b) student awareness, **c)** Institutional policy.

Also shown in Table 3 and Fig 4, are important limitations regarding the awareness of AI and relevant educational policies. For example, only 43.59% of responders stated that they were “very familiar” with LLMs, with the remaining “somewhat familiar” (46.15%) and “not familiar” (10.26%). Similarly, only 22.22% of responders stated that their students were “highly aware” of the implications of using AI tools in their coursework, with the remaining “somewhat aware” (65.81%) and “not aware” (11.97%). Moreover, students’ lack of awareness may likely be related to the fact that only 47.86% of responders reported that their institutions had policies regarding the use of AI in assessments. Such results suggest a need for improved awareness for both staff and students, as well as formal policies to ensure that staff and students are aware of the rules and regulations surrounding the use of AI in education.

**Fig 5:**
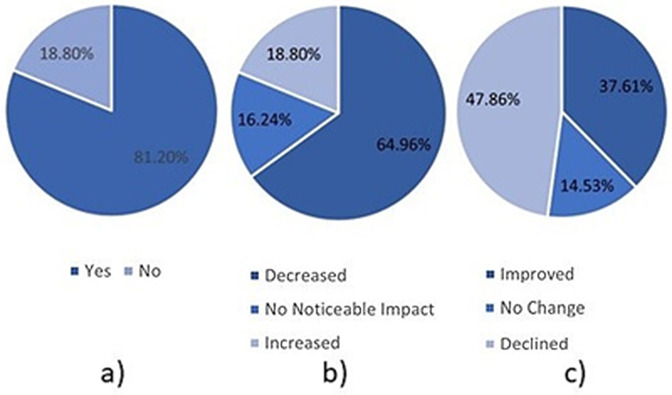
Pie charts demonstrating the impacts of AI in education. **a)** AI identified, b) effect on originality, c) effect on quality.

**Fig 6:**
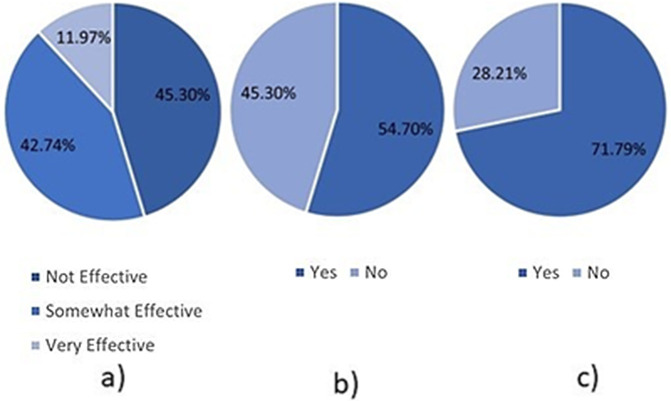
Pie charts describing academics’ opinions regarding AI in education. a) identification effectiveness, b) should AI be allowed, c) would autonomous assessment be beneficial?.

Moreover, Table 3 and Fig 5 further highlights the importance of AI awareness and policies. For example, 81.20% of responders reported identifying evidence of AI use in student assessments. Moreover, 64.96% of responders reported that AI usage reduced assessment originality, and 47.86% also stated that it negatively affected the quality of student work. Furthermore, as shown in Fig 6 c) only 11.97% of responders reported that their institution was effective at identifying AI-generated content in student work, with the remainder stating that their institutions were somewhat effective (42.74%) or not effective (45.30%). These statistics are concerning because they suggest that academic standards may be threatened, and institutions may not be well-equipped to handle the current situation.

Furthermore, Table 3 and Fig 6 describes academics’ views regarding the use of AI in education. Despite the aforementioned issues, academics tend to have positive opinions of AI in education. For example, 54.70% of responders stated that they believed AI should be allowed in assessments, whereas 71.79% (Fig 6-C) indicated they felt that AI-based autonomous assessment would benefit them.

Table 4 shows the statistical significance of the dependence between various factors (i.e., questions asked) and experts’ opinions on the acceptability of GAI for students’ work submission and for its use in automated assessment (i.e., proposed framework). It can be noted that the acceptability of GAI is significantly dependent on most factors (i.e., p = 0.05) except detecting AI-generated content (p = 0.07), policies (p = 0.48), and awareness (p = 0.07). Furthermore, the acceptability of GAI for students’ work submissions is significantly dependent upon familiarity with AI-based tools (p = 0.02). The survey results show that 67% of responses from students with high familiarity with LLMs indicate acceptance of their use. On the other hand, it decreased to 25% only among participants who are unfamiliar with AI-based tools.

**Table 4:** Interdependence between the recommendation of AI-based auto assessment tools, acceptance of the usage of AI-based tools by students, and other related factors.

	GAI for auto-assessment			Allow GAI		
	*χ*2	df	*p*-value	*χ*2	df	*p*-value
LLM-Familiarity	0.39	2	0.82	7.7	2	0.02
ChatGPT noticed	0.46	1	0.49	6.91	1	0.008
Originality affected	5.35	2	0.06	12.02	2	0.002
Quality affected	2.14	2	0.34	15.68	2	0.0003
Detecting AI-generated content	0.39	2	0.81	0.60	2	0.73
Allow-GAI	7.31	1	0.006	7.31	1	0.006
Policies	0	1	1	0.48	1	0.48
Awareness	1.52	2	0.46	5.09	2	0.07

Regarding the use of GAI for automated grading and assessment, no significant relationship is noticed mostly (p > 0.05) except ‘allow-GAI’, which indicates strong interdependence (p=0.006). In other words, regardless of familiarity, awareness, and other factors (Table 4), the participants recommend using AI-based autonomous assessment (i.e., the proposed framework). Furthermore, the distribution of responses indicates that a high proportion (83%) of the staff who accept the use of AI in students’ work also recommend using AI-based tools for automated assessment (i.e., proposed idea). However, this opinion decreased to 58% for participants who believed students should not use GAI in their submissions.

These statistics clearly support the argument in RQ2 that AI-based tools would be useful for automating the grading of students’ work submissions based on their understanding and knowledge of the subject matter, instead of detecting ChatGPT (GAI) usage (which is the current standard practice). The statistics also support the concept of allowing students to utilise GAI for the assessments (e.g., report writing, coursework, etc.) while new policy standards would be required to do so.

As shown in Table 3, 81.20% of the academic staff surveyed reported noticing GAI in students’ assessments. However, only 10.26% of those surveyed reported being very familiar with LLMs. Therefore, training is required in established detection techniques, such as those described in [49].

Similarly, only 11.97% stated that students were highly aware of the implications of GAI in assessments. Recent works such as [50] have investigated students’ perceptions of AI usage and found that it is popular. However, they have not investigated the awareness of potential implications, such as academic misconduct. Therefore, there is a clear need to ensure that all students are familiar with current policies and the implications of unethical GAI usage.

Despite the prevalence of GAI, many academics consider a complete ban on GAI necessary to maintain academic standards [51]. However, the questionnaire results suggest that both academics and students find benefits from such technology, benefits that would be lost under a total ban. Moreover, existing works, such as [50], suggest that students can ethically use tools such as ChatGPT to support their studies.

**Fig 7:**
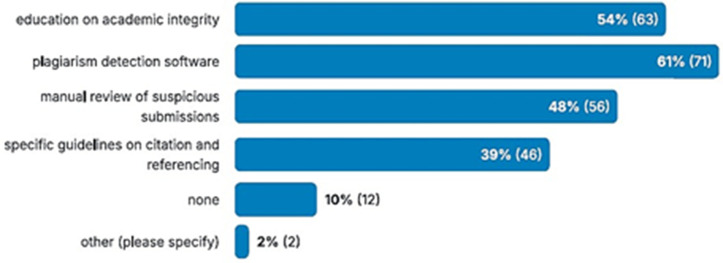
Responses to the question “What strategies, if any, does your institution employ to mitigate the impact of AI tools on assessment integrity? (Check all that apply)”.

However, there is a lack of consistent policy on the use of AI in academia, particularly regarding assessment. As shown in Fig 7, there is no clear single policy. A broad range of policies is reported, and 10% of respondents report that no policy is employed in their institution. Similarly, as reported in Table 3, only 47.86% of respondents stated that their institution had specific policies related to AI usage. This is supported by works such as [52], which highlights a lack of clear and consistent policies. Therefore, there is a clear need for consistent policies in academia. However, such policies must be robust and promote the ethical use of AI. As shown in Table 3, 54.70% of academic respondents favour allowing students to use AI. Therefore, it is unlikely that academics would desire a complete ban.

Moreover, academics also see the potential benefits of AI for themselves. For example, Table 3 shows that 71.79% of respondents would benefit from assessing students and providing feedback. Therefore, there is evident demand for an automated assessment framework, one we propose in Section 5.

### 4.2. Open-ended question analysis

In addition to analysing the MCQs, responses to the open-ended questions have also been analysed. The open-ended questions described in Section 3 were analysed using LDA [51] to identify common topics and keywords. Two open-ended questions are provided in Section 3. The first addresses challenges academics have faced, and the second asks academics to provide suggestions to improve assessments in the presence of AI tools.

LDA was chosen for its ability to uncover latent thematic structures in qualitative responses. Topics were identified based on coherence scores and interpretability by the research team. However, it is important to acknowledge that topic modelling depends on response length and quality, and some nuanced themes may be underrepresented.

*“What challenges, if any, have you encountered in assessing students’ work in the presence of AI tools like ChatGPT?”*

In response to the above question, several keywords suggest common challenges. For example, “detection”, “tool”, “understanding”, and “originality” are amongst the most common keywords. Identifying the common keywords in the original responses highlights common challenges, such as a lack of originality, as already highlighted in Table 3. Additionally, it highlights additional concerns related to assessing students’ understanding in the presence of tools such as ChatGPT. Similarly, many academics wish for better tools to detect GAI usage.

*“Do you have any suggestions for improving the assessment process in light of the prevalence of AI tools like ChatGPT?* A plethora of keywords is presented in response to the request for suggestions*.* For example, common keywords include *“guideline”, “policy”, “viva”, “presentation”, “face [to face]”, “written”, “practical”, “diversify”, and “exam”.* Moreover, further investigation of the suggestion shows a common theme of alternative assessment. For example, many responses suggest alternative and diverse assessments to prevent the use of AI in assessments, such as presentations, oral responses, exams, and practical. Another common suggestion is to create and implement clear AI guidelines and policies.

Table 5 summarises the topics identified from the open-ended responses using LDA, providing a structured overview of the thematic content and highlighting differences across countries. Four topics were extracted, each accompanied by a descriptive label and the ten most representative keywords. Overall coherence for the four-topic model was 0.58, reflecting moderate interpretability and semantic clarity. Mean topic prevalence across all responses ranged from 0.23 to 0.29, indicating that academics often expressed multiple concerns concurrently rather than focusing on a single issue. The topics capture both practical and conceptual dimensions of AI use in higher education: detection challenges (Topic 0), student use of AI in assignments (Topic 1), operational and software-related difficulties (Topic 2), and potential impacts on student learning and assessment validity (Topic 3).

**Table 5:** Topics Identified in Open-Ended Responses Using LDA, with Country-Specific Prevalence and Effect Sizes.

Country	Topic	Label	Representative Keywords	Mean Prevalence (Country)	Mean Prevalence (Other)	Cohen’s h
Iraq	0	Detection challenges	student, used, AI, know, ChatGPT, easy, sentence, hard, time, dont	0.25	0.21	0.09
Iraq	1	Student use of AI	student, use, ai, tool, ChatGPT, assignment, work, dont, make, correct	0.26	0.31	−0.11
Iraq	2	Practical challenges	use, ai, student, used, work, tool, hard, software, writing, need	0.24	0.23	0.02
Iraq	3	Impact on learning & assessment	ai, work, lack, student, understanding, generated, assessing, tool, content, text	0.24	0.23	0.01
UAE	0	Detection challenges	student, used, ai, know, ChatGPT, easy, sentence, hard, time, dont	0.12	0.25	−0.32
UAE	1	Student use of AI	student, use, ai, tool, ChatGPT, assignment, work, dont, make, correct	0.42	0.26	0.33
UAE	2	Practical challenges	use, ai, student, used, work, tool, hard, software, writing, need	0.19	0.24	−0.11
UAE	3	Impact on learning & assessment	ai, work, lack, student, understanding, generated, assessing, tool, content, text	0.25	0.23	0.03
UK	0	Detection challenges	student, used, ai, know, ChatGPT, easy, sentence, hard, time, dont	0.25	0.22	0.07
UK	1	Student use of AI	student, use, ai, tool, ChatGPT, assignment, work, dont, make, correct	0.25	0.31	−0.13
UK	2	Practical challenges	use, ai, student, used, work, tool, hard, software, writing, need	0.26	0.22	0.09
UK	3	Impact on learning & assessment	ai, work, lack, student, understanding, generated, assessing, tool, content, text	0.23	0.24	−0.02

Beyond descriptive keywords, the table shows per-country variation in topic prevalence and the corresponding Cohen’s h effect sizes. Most differences between countries were modest, though some patterns emerged: for example, respondents from the UAE emphasised Topic 1 (“Student use of AI”) more than other countries (h = 0.33), while UK respondents slightly emphasised Topic 2 (“Practical challenges”) relative to other countries (h = 0.10). These results suggest that while underlying concerns about AI in assessment are broadly shared, the national context can subtly influence which aspects of the issue are most salient to academics. Overall, the combined analysis of representative keywords, prevalence, and effect sizes provides a nuanced, statistically grounded understanding of the qualitative data, complementing the initial descriptive analysis.

### 4.3. Qualitative validation of proposed framework

The responses reported in Table 3 suggest a clear need for the proposed framework presented in Fig 2. For instance, 81.20% of respondents reported noticing LLM usage in student work, and 64.96% reported a negative impact on originality, with 47.86% also suggesting that the overall quality of work was reduced. However, 56.41% indicated that they were not very familiar with LLMs, and 88.03% were not very effective at spotting AI-generated content, highlighting the need for assistance in assessing students’ work, in addition to their knowledge and understanding of the work that they have submitted.

Moreover, 54.70% of respondents indicated that they believed that GAI content should be allowed in academia. Therefore, necessary mechanisms are required to ensure that students still gain appropriate knowledge and skills while using GAI. Thus, the proposed framework may be used to support policies that promote the ethical use of GAI content by examining the students’ knowledge of the content in a time-controlled manner.

### 4.4. Framework evaluation results: Student and instructor feedback

To complement the survey-based findings and provide a practical demonstration of the proposed framework, a prototype application was developed and evaluated. The prototype implements the core components of the autonomous assessment system, including document submission, AI-generated multiple-choice question creation, time-controlled interaction and automated feedback generation. As shown in Table 6, the functional testing and preliminary evaluation were conducted under controlled conditions to determine whether the system operates as intended.

**Table 6:** Functional validation outcomes of the prototype auto-assessment framework.

Test Cases	Description	Expected Result	Actual Result	Pass/ Fail
**File Upload**	Upload valid.txt,.pdf and.docx file	File accepted; text extracted	File accepted; text extracted	✔
**File Upload (Unsupported File Format)**	Upload a.png file	File not accepted; error message	Exception raised; file not accepted	✔
**Character Limit**	Upload.pdf over 7000 characters	User prompted to try new file; file not accepted	User prompted to try new file; file not accepted	✔
**MCQ Generation**	Generate questions from valid document	10 MCQs with 4 valid options	10 MCQs with 4 valid options generated successfully in under 20 seconds	✔
**Incomplete Model Output**	Trigger incomplete output (small text input)	Iterative retry mechanism triggered	Iterative retry mechanism triggered	✔
**Quiz Navigation**	Navigate through questions using buttons	Buttons work correctly	Buttons work correctly; navigation works	✔
**Quiz Submission**	Submit quiz with all questions answered	Score is shown on results screen	Score and result display correctly	✔
**Incomplete Submission**	Submit quiz with unanswered questions	Submission not accepted; user prompted to answer question	User prompted to answer incomplete question; submission not accepted	✔
**Feedback Display**	Click “provide feedback” button	Questionnaire opens	Questionnaire opens; Feedback displayed correctly	✔
**Feedback Save**	Submit Feedback form	Data saved to.csv successfully	CSV file created and data saved successfully	✔

The functional testing results in Table 6 indicate that the prototype application performed reliably across all defined test scenarios. Core functionalities, including document upload, question generation, assessment navigation, response submission and results presentation, were successfully executed in all cases. The system consistently produced the expected outputs and appropriately handled incomplete or invalid interactions through user prompts. These tests were conducted in the presence of seven experienced academics and confirm that the prototype operates as intended at a functional level.

**Fig 8:**
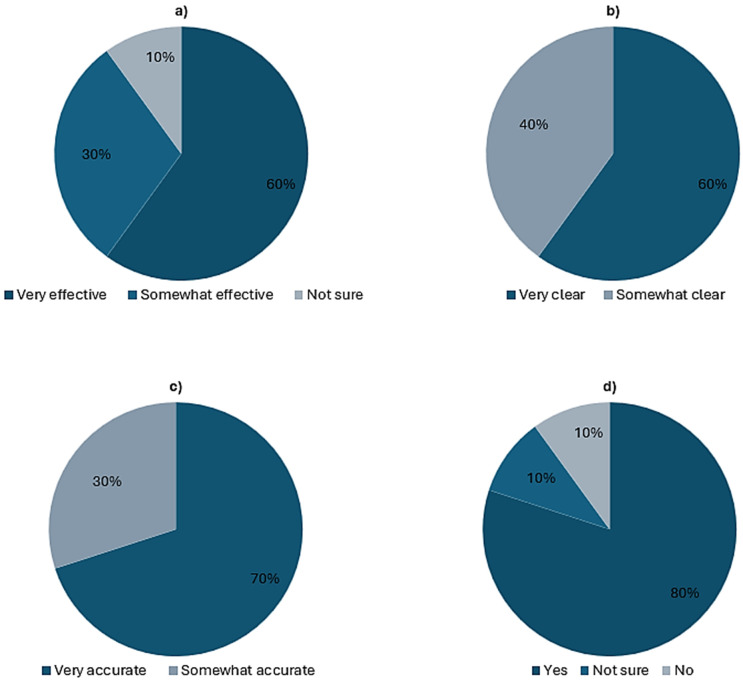
Visualising responses to prototype evaluation questions. a) effectiveness of AI-generated MCQs (Q5); b) clarity and understandability of AI-generated MCQs (Q7); c) accuracy of AI-generated feedback (Q8); d) feasibility of the application for use in academic institutions (Q9).

In addition to functional testing, we performed qualitative evaluation (shown in Table 7) to capture participant perceptions of our prototype’s usability, reliability, and acceptability in the context of future academic assessment practices. The evaluation results reflect generally positive perceptions of the prototype application. Most participants (70%−100%) reported that the system was easy to understand and navigate, and the majority responded (see Fig 8) that the AI-generated questions as clear and relevant to the submitted content. Most importantly, the participants indicated that the feedback and grading outcomes were appropriate representations of their performance.

**Table 7:** Evaluation results of the prototype application based on participant feedback (n = 20).

Question	Response Options	Most Common Response		Response Distribution N = 20
Q1: How familiar are you with using AI tools like ChatGPT in educational settings?		Very familiar, Somewhat familiar, Not familiar at all	Somewhat familiar	6 Very, 10 Somewhat, 4 Not familiar
Q2: Have you faced any challenges while using ChatGPT or similar AI tools for learning or assessments?		Yes, No	No	6 Yes, 14 No
Q3: How aware are you of the policies and guidelines surrounding the use of AI tools in education?		Very aware, Somewhat aware, Not aware at all	Somewhat aware	4 Very, 12 Somewhat, 4 Not aware
Q4: What concerns, if any, do you have about using AI for academic purposes?		Open-ended	N/A (open text)	Common themes included: bias, misuse, cheating, lack of clarity
Q5: How effective was the MCQ generation by the application based on the provided document?		Very effective, Somewhat effective, Not effective, Not sure	Very effective	12 Very, 6 Somewhat, 2 Not sure
Q6: How easy was it to navigate the interface of the application?		Very easy, Somewhat easy, Difficult, Very difficult	Very easy	14 Very, 6 Somewhat
Q7: How clear and understandable were the AI-generated MCQs?		Very clear, Somewhat clear, Not clear at all	Very clear	12 Very, 8 Somewhat
Q8: How accurate was the feedback (validation of your responses)?		Very accurate, Somewhat accurate, Not accurate at all	Very accurate	14 Very, 6 Somewhat
Q9: Is the application a feasible solution for academic institutions?		Yes, No, Not sure	Yes	16 Yes, 2 Not sure, 2 No
Q10: What suggestions do you have for improving the application?		Open-ended	N/A (open text)	Themes: UI style, more colour, add explanations

The outcomes related to policy awareness in Table 7 are also aligned with our survey results (in Table 3, Fig 4 in Section 4.1) where participants raised the concerns related to awareness of misuse of AI and the need for clearer institutional guidelines. It can also be noticed that only 10% of the participants responded negatively about the feasibility of the proposed assessment framework (Fig 8. d) which reflects the outcomes of our survey (Fig 6.c) showing majority (72%) with positive response. Overall, the outcomes suggest strong initial acceptance of the prototype as an early-stage implementation of the proposed framework.

### 4.5. Comparison with existing solutions

A comparison of related solutions is provided in Table 8. Like the proposed framework, [53] and [54] provide students with real-time feedback based on their answers to MCQs. However, these works use fixed question banks and, therefore, cannot tailor questions to students, unlike the proposed method, which generates custom question banks for each student. Alternatively, both [55] and [56] use AI to predict a score and to suggest feedback for the work, using Natural Language Processing (NLP) and Deep Learning (DL), respectively. However, both works would require teachers to confirm results and provide feedback. However, neither method can provide granular knowledge of students’ understanding (e.g., strengths and weaknesses related to subtopics and questions).

**Table 8:** A comparison of existing automated assessment platforms regarding their features and characteristics. Including the use of AI in the platform (if any), the academic subject (ALL if it is subject-independent), misconduct verification checks, real-time feedback, whether unique questions are used for each student, adaptive testing, and time-limited assessment functionality.

Ref	AI	Subject	Interactive	Understanding Verification	Verification Checks		Real-Time Feedback	Unique Question Bank	Adaptive Testing	Time-Limited Assessment
					Plagiarism	GAI				
[53]	No	Chemistry	✔	✔	✘	✘	✔	✘	✘	✔
[54]	No	All	✔	✔	✘	✘	✔	✘	✘	✘
[55]	NLP	Maths	✘	✘	✘	✘	✘	✘	✘	✘
[56]	DL	English	✘	✘	✘	✘	✔	✘	✘	✘
**Ours**	**GAI**	**All**	✔	✔	✔	✔	✔	✔	✔	✔

Furthermore, with the exception of our proposed framework, none of the works reviewed in Table 8 provide verification checks, such as for plagiarism or unethical use of GAI. Specifically, existing works do not assess students’ knowledge of their own work, making it impossible to detect academic misconduct such as plagiarism or GAI misuse. Similarly, none of the works uses adaptive testing to tailor assessments to individual students, again limiting the degree to which students’ understanding, particularly in relation to individually submitted work, is assessed. Moreover, only our proposed framework and [53] provide facilities for time-limited assessments. Unlike the works presented in Table 5, the proposed method assesses the understanding and knowledge of the work that they submitted, thus tailoring assessments while mitigating misconduct issues.

To conclude, the practical implementation of the proposed framework involves several challenges that must be acknowledged. Our survey highlights a notable lack of familiarity with AI tools among academics, which could hinder effective adoption without targeted training and support. Additionally, integrating this framework within diverse institutional contexts requires careful consideration of local academic policies, technological infrastructure, and pedagogical approaches. While the framework offers adaptive and autonomous assessment capabilities, further pilot testing and iterative refinement are necessary to validate its effectiveness in real-world educational settings. Addressing these implementation aspects is crucial to ensure the framework’s sustainability and alignment with institutional goals.

## 5. Conclusion and future work

This study has explored the opportunities and challenges posed by GAI in higher education assessments, drawing insights from a survey of academics across three countries. The findings highlight a clear gap between educators’ optimistic attitudes towards AI integration and the current lack of institutional policies, training, and tools to manage AI-generated content in assessments. Despite concerns about originality and assessment integrity, most academics oppose outright bans and instead advocate for policy development, staff training, and innovative assessment strategies.

To address these needs, we introduced a new framework for automated assessment along with human oversight, designed to interactively and ethically assess students’ understanding. Unlike existing systems, the framework incorporates adaptive testing, dynamically generated question banks, and real-time feedback while embedding verification mechanisms to detect AI misuse.

In addition, the topic modelling analysis using LDA provided a structured, data-driven view of academics’ open-ended responses. By identifying common themes, representative keywords, and variations in topic prevalence across countries, this analysis complemented the quantitative survey findings and highlighted nuanced patterns in perceptions of AI in assessment. Future work could extend this approach by incorporating larger, more diverse datasets, enabling cross-institutional and longitudinal comparisons, and potentially integrating LDA outputs into the design of adaptive assessment frameworks to better align evaluation strategies with emerging AI practices.

Considering these findings, we recommend a collaborative approach to policy development that aligns technological advancements with academic integrity. Stakeholder engagement, particularly with academics, students, and policymakers, will be essential to develop balanced guidelines that enable the ethical use of AI while maintaining rigorous assessment standards. Moreover, following the survey outcomes, implementation and initial evaluation of the proposed automated assessment framework, future work should focus on its broader integration and longitudinal evaluation within the academic sector. The results presented in Section 4 indicate strong academic support for such a system, and wider deployment will allow further validation and refinement across diverse educational contexts.

Finally, we acknowledge that the sample size and geographic scope of this study, while sufficient for exploratory analysis, limit the generalisability of the findings. Future research should aim to involve a larger number of participants across a broader range of countries and educational contexts to validate and expand upon the patterns identified in this study. Expanding the dataset would allow for a more comprehensive understanding of institutional responses to generative AI across diverse higher education systems.

## Supporting information

S1 Walkthrough Step-by-step system walkthrough with annotated snapshots illustrating the workflow and procedures described in the study. (PDF)

S1 Dataset Dataset file. (CSV)

S1 File Inclusivity in Global Research. (DOCX)
