# Supplementary material for: Auto-assessment of assessment: A human-in-the-loop AI framework addressing policy gaps in academic assessment
Source: PLoS One. 2026 Apr 15;21(4):e0346815. doi: 10.1371/journal.pone.0346815 (PMC13082603; doi:10.1371/journal.pone.0346815)
Supplement: S1 Walkthrough — (PDF) [file pone.0346815.s001.pdf]

This document presents system walkthrough diagrams illustrating the operational workflow of the prototype application developed to demonstrate the practical feasibility of the proposed autonomous assessment framework. The diagrams provide a step-by-step overview of system components, user interactions and system responses across the main stages of the assessment process used in functional testing.

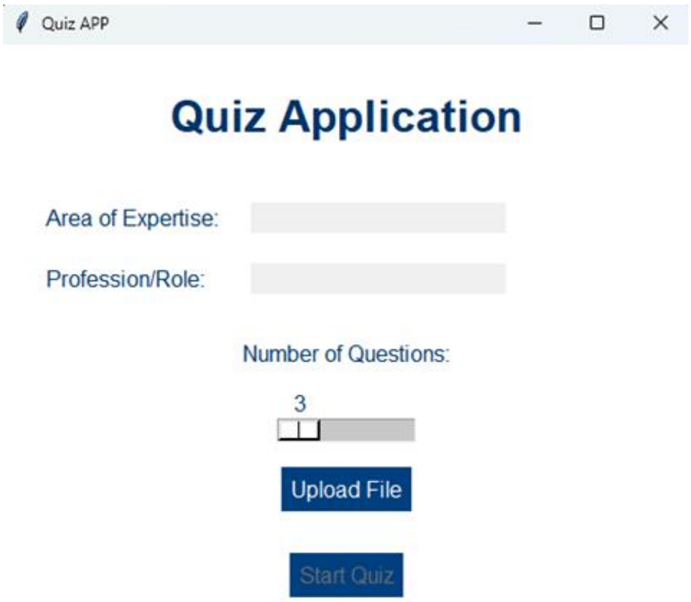

Figure A1. Prototype application interface for document submission.

This screen allows the user to upload their assessment document for analysis by the system.

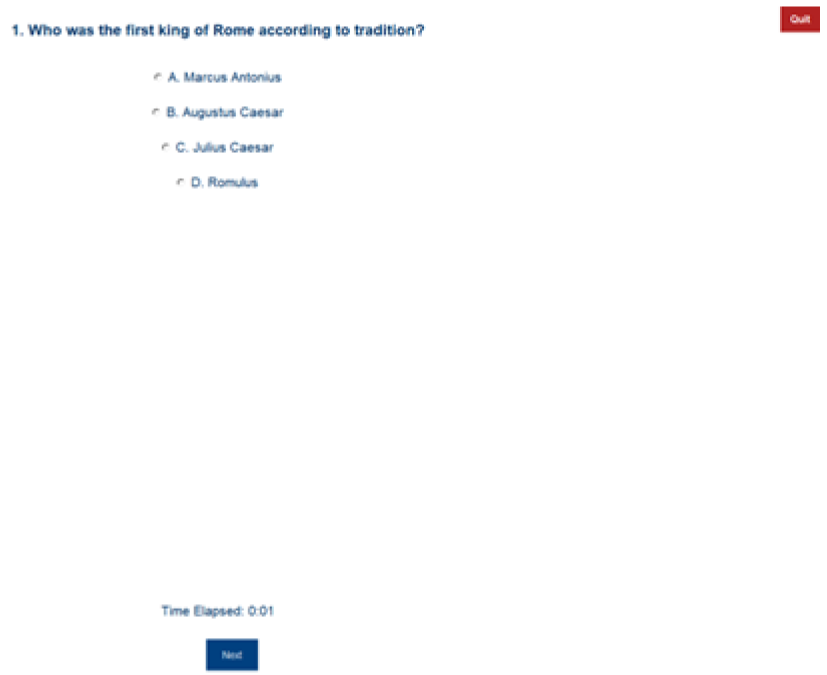

Figure A2. AI-generated assessment question interface.

This screen presents the first question based on the user-submitted document.

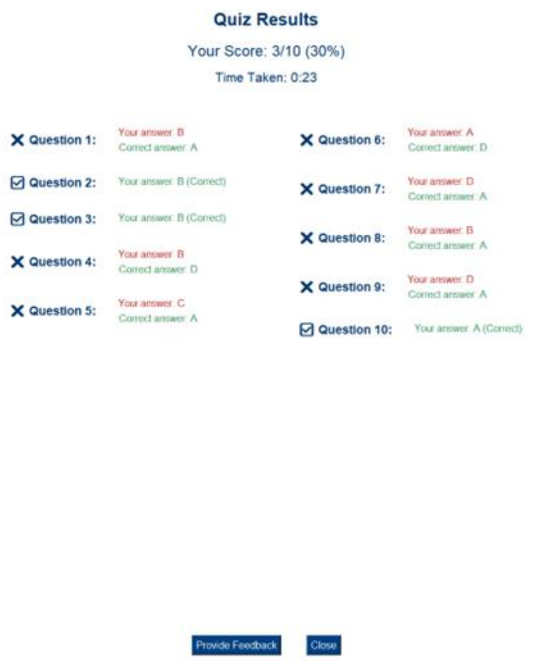

Figure A3. Prototype results interface.

This screen displays the system-generated assessment outcome following user response
